# Characterization of Microtubule-Binding and Dimerization Activity of *Giardia lamblia* End-Binding 1 Protein

**DOI:** 10.1371/journal.pone.0097850

**Published:** 2014-05-14

**Authors:** Juri Kim, Sara Nagami, Kyu-Ho Lee, Soon-Jung Park

**Affiliations:** 1 Department of Environmental Medical Biology and Institute of Tropical Medicine, Brain Korea 21 PLUS Project for Medical Science, Yonsei University College of Medicine, Seoul, South Korea; 2 Department of Life Science and Interdisciplinary Program of Integrated Biotechnology, Sogang University, Seoul, South Korea; University at Buffalo, United States of America

## Abstract

End-binding 1 (EB1) proteins are evolutionarily conserved components of microtubule (MT) plus-end tracking protein that regulate MT dynamics. *Giardia lamblia*, with two nuclei and cytoskeletal structures, requires accurate MT distribution for division. In this study, we show that a single EB1 homolog gene of *G. lamblia* regulates MT dynamics in mitosis. The haemagglutinin-tagged *G. lamblia* EB1 (GlEB1) localizes to the nuclear envelopes and median bodies, and is transiently present in mitotic spindles of dividing cells. Knockdown of GlEB1 expression using the morpholinos-based anti-EB1 oligonucleotides, resulted in a significant defect in mitosis of *Giardia* trophozoites. The MT-binding assays using recombinant GlEB1 (rGlEB1) proteins demonstrated that rGlEB1_102–238_, but not rGlEB1_1–184_, maintains an MT-binding ability comparable with that of the full length protein, rGlEB1_1–238_. Size exclusion chromatography showed that rGlEB1 is present as a dimer formed by its C-terminal domain and a disulfide bond. *In vitro*-mutagenesis of GlEB1 indicated that an intermolecular disulfide bond is made between cysteine #13 of the two monomers. Complementation assay using the *BIM1* knockout mutant yeast, the yeast homolog of mammalian EB1, indicated that expression of the C13S mutant GlEB1 protein cannot rescue the mitotic defect of the *BIM1* mutant yeast. These results suggest that dimerization of GlEB1 via the 13th cysteine residues plays a role during mitosis in *Giardia*.

## Introduction

Microtubules (MTs) are dynamic polymers involved in mitosis, organelle biogenesis, and intracellular transport [1]. Growth and catastrophe of MTs are mediated by MT-associated proteins including plus-end tracking proteins (+TIPs) which preferentially bind to the growing ends of polymerized MTs *in vivo* [2]. Among the +TIPs, end-binding 1 (EB1) proteins are the main regulator of the protein complexes at the plus end, as shown in mammalian EB1 [3] and yeast EB1 homologs, Mal3p [4] and Bim1p [5].

The proteins belonging to the EB1 family are composed of an N-terminal calponin homology (CH) domain, a composite domain consisting of a α-helically coiled-coli and a unique EB1 homology region, and the C-terminal acidic tail ending with an EEY/F motif [6]. The CH domain is reported to be involved in MT binding [4], whereas the EB1domain play a role in dimerization as well as binding to the EB1-interacting proteins [7]. The EEY/F motif serves as a binding site for p150^Glued^ in mammals [8]. The EB1 protein is one of highly conserved proteins among in all eukaryotes, including *Giardia lamblia*, a protozoan belonging to the earliest diverging eukaryotic lineage [9].

*G. lamblia* has two nuclei and cytoskeletal structures including an adhesive disc, a median body, and four pairs of flagella [10]. Observations using three-dimensional deconvolution and electron microscopies indicated that two extranuclear spindles move chromosomes laterally through a polar opening in the nuclear membrane during cell division of *G. lamblia* [11]. Using a dominant-negative mutant, kinesin-13 was found to control MT dynamics in *G. lamblia* [12]. A single open reading frame (ORF) encoding an EB1-homologous protein was found in the database of *G. lamblia*. Using GFP-tagged EB1, *G. lamblia* EB1 (GlEB1) was found at the flagellar tips and median bodies [12]. In addition, the role of GlEB1 was assessed by complementation assays using a *BIM1* mutant of *Saccharomyces cerevisiae*, in which proper positioning of the nucleus is abolished [13]. Through co-immunoprecipitation and yeast two-hybrid assays, block of proliferation 1 and cytoskeletal proteins such as β- and γ-giardins, were identified as EB1-interacting proteins [14], [15], [16]. However, it remains to be elucidated how GlEB1 modulates MT dynamics during the cell cycle of *G. lamblia*.

In this study, expression and intracellular localization of GlEB1 were examined in various stages using a transgenic *G. lamblia* expressing haemagglutinin (HA) epitope-tagged EB1. In addition, a biochemical characterization of GlEB1 was performed by defining the domains and an amino acid residue responsible for MT binding and dimerization.

## Materials and Methods

### *Giardia* cell culture

Trophozoites of the *Giardia* WB strain (ATCC 30957; Table 1) were grown for 72 h in a normal TYI-S-33 medium (2% casein digest, 1% yeast extract, 1% glucose, 0.2% NaCl, 0.2% L-cysteine, 0.02% ascorbic acid, 0.2% K_2_HPO_4_, 0.06% KH_2_PO_4_, 10% calf serum, and 0.75 mg/mL bovine bile, pH 7.1) [17].

**Table 1:** Strains and plasmids used in this study.

Organism/Plasmid	Description	Source/reference
*G. lamblia*		
ATCC 30957	Clinical isolation	ATCC
*E. coli*		
DH5α	*supE44, ΔlacU169 (Φ80 lacZ ΔM15), hsdR17, recA1, endA1, gyrA96, thi-1, relA1*	Invitrogen
BL21 (DE3)	*F′, ompT, hsdS_B_(r_B_^-^m_B_^-^) gal, dcm (DE3)*	Invitrogen
*S. cerevisiae*		
W303a	MATa, *ade2-1, ura3-1, trp1-1, leu2-3, 112 his3-11, 15 can1-100*	[13]
YSK1134	W303a, *Δbim1::Kan* R, *Δbfa1:: HIS3, [pRS304-BFA1-TAP]*	[13]
Plasmids		
pLop2	Shuttle vector, Amp^R^	[24]
pNLop2-GItetR	*neo* gene	[24]
pLop2-eb1-HA	pLop2, 817 bp of *G. lamblia eb1* promoter and *eb1* (GiardiaDB; GL50803_14048)	This study
pNLop2-eb1-HA-GItetR	*neo* gene, 817 bp of *G. lamblia eb1* promoter and *eb1*	This study
pET28b	Expression vector, Kan^R^	Novagen
pET28b-γ-tubulin	pET28b, 1478 bp encoding *G. lamblia γ-tubulin* (GiardiaDB; GL50803_114218)	This study
pET21b	Expression vector, Amp^R^	Novagen
pET21b-EB1-Full	pET21b, 717 bp encoding *G. lamblia eb1*	This study
pET21b-EB1-CHD	pET21b, 552 bp encoding *G. lamblia eb1*	This study
pET21b-EB1-EBD	pET21b, 474 bp encoding *G. lamblia eb1*	This study
pET21b-EB1-C13S	pET21b, 717 bp encoding *G. lamblia C13S eb1*	This study
pET21b-EB1-C46S	pET21b, 717 bp encoding *G. lamblia C46S eb1*	This study
pRS426+P_GAL1-10_	pRS426, *GAL1-10* promoter	[13]
pRS426+P_GAL1-10_-EB1-C13S	pRS426+P_GAL1-10_, 717 bp encoding *G. lamblia C13S eb1*	This study

Amp, ampicillin; Kan, kanamycin;

R , resistant; DNA-BD, DNA binding domain; AD-activation domain; HA, haemagglutinin.

To induce encystation *in vitro*, *G. lamblia* trophozoites were transferred into an encystation medium (TYI-S-33 medium, 10 mg/mL bovine bile, pH 7.8) [18]. At various time-points after the incubation in the encystation medium, the cells were harvested by centrifugation at 3000 rpm for 15 min at 4°C. To monitor the encystation process, intracellular level of CWP1 [19] was measured in the harvested cells.

### Construction of *G. lamblia* expressing HA Epitope-tagged GlEB1

Plasmid pLop2 and pNLop2-GItetR were a gift from Dr. Jung-Hsiang Tai [20]. To generate an HA epitope tag to the C-terminal of the *eb1* gene, a 950 bp DNA fragment containing the promoter and the full ORF of the *eb1* gene was amplified from *G. lamblia* WB genomic DNA by PCR using two primers, eb1-NcoI-F and eb1-HA-R (Table 2). NcoI and EcoRI sites, located at the ends of the resultant *eb1* DNA, were used for cloning into the corresponding site of plasmid pLop2, resulting in the plasmid pLop2-eb1-HA. A 950 bp NheI/SalI fragment of pLop2-eb1-HA was cloned into the plasmid pNLop2-GItetR to yield the plasmid pNLop2-eb1-HA-GItetR, in which GlEB1 is expressed as a fused protein in frame with an HA-epitope. All constructs were verified by DNA sequencing provided by a sequencing service company (Macrogen, Seoul, Korea).

**Table 2:** Oligonucleotides used in this study.

Name	Nucleotide sequences(5′-3′)
For the construction of HA epitope tagged GlEB1	
eb1-NcoI-F	GCATCCATGGTGCCATCTGTACCACAATC
eb1-HA-R	GCGAATTCTTACGATTCATCAGCGTAATCTGGTACGTCGTATGGGTACTCCTGATGATACTCCGCA
For the construction of recombinant *G. lamlbia* γ-tubulin	
γ-tubulin-F	CATGCCATGGGCATGTGCGTTTATATTGAA
γ-tubulin-R	CCGCTCGAGCATCCCGATATATACTCAAG
For the construction of recombinant GlEB1 proteins	
EB1-full-ER1-F	GCGAATTCGATGCCGCCGGTAAAAGCACC
EB1-CHD-R	CCGCTCGAGCTCTAGCTGACCCTTTGCAAT
EB1-EBD-F	GCGAATTCGTACATGGACAACTTCGAG
EB1-full-Xho1-R	CCGCTCGAGCTGATGATACTCCGCATACA
For the construction of site-directed mutant GlEB1 proteins	
EB-C13S-21b-F	GCGAATTCGATGCCGCCGGTAAAAGCACCCGGAAATGTGTCTGAC***AGC***TACTTTGTA
EB-C46S-21b-F	CACCACTAT***TCA***ATGGCCCTG
EB-C46S-21b-R	CAGGGCCAT***TGA***ATAGTGGTG
Morpholinos sequence	
Anti-EB1	TTCCGGGTGCTTTTACCGGCGGCAT
Mispair EB1	TTCCGG***C***T***C***CTTTTA***G***CG***C***C***C***GCAT

Restriction enzyme sites are underlined.

Mutation sites are indicated in bold and italic letters.

The trophozoites were grown for 72 h in normal TYI-S-33 medium. Thirty micrograms of pNLop2-eb1-HA-GItetR were transformed into 1×10^7^ trophozoites by electroporation under the following conditions: 350 volts, 1000 µF, and 700 Ω (BioRad). Trophozoites harboring pNLop2-eb1-HA-GItetR were selected by adding G418 (Life Technologies) to the TYI-S-33 medium at a final concentration of 150 µg/mL, and after 4 to 5 days of cultivation, the resistant cells were transferred to and maintained in the medium containing 600 µg/mL of G418. As a control, trophozoites carrying pNLop2-GItetR were constructed as described above.

### Western Blot Analysis

Cell extracts were prepared from *G. lamblia* containing pNLop2-GItetR, or pNLop2-eb1-HA-GItetR in a phosphate buffered saline (PBS: 137 mM NaCl, 2.7 mM KCl, 10.1 mM Na_2_HPO_4_, and 2 mM KH_2_PO_4_, pH 7.4), separated by SDS-PAGE, and transferred onto a polyvinylidenefluoride (PVDF) membrane (Millipore). The membrane was incubated with monoclonal mouse anti-HA (1∶2000; Sigma) in a blocking solution [Tris-buffered saline with Tween 20 (TBST); 50 mM Tris-HCl, 5% skim milk, and 0.05% Tween 20] at 4°C overnight. Following incubation with horseradish peroxidase (HRP)-conjugated secondary antibodies, the immunoreactive protein was visualized using an enhanced chemiluminescence (ECL) system (Amersham Pharmacia). Membranes were incubated in a stripping buffer (Thermo Scientific) at room temperature for 30 min, and then reacted with polyclonal rat antibodies specific to the α-tubulin of *G. lamblia* (1∶10000) [21].

In the case of trophozoites with pNLop2-eb1-HA-GItetR, they were prepared under various cell cycle stage: without aphidicolin treatment, 6 h-aphidicolin treatment, or released from the aphidicolin treatment every hour up to 6 h. Intracellular levels of GlEB1 were monitored in these cells by Western blot analysis using anti-HA antibodies (1∶2000). As a loading control, an amount of α-tubulin was also detected in these cell extracts using anti-Glα-tubulin antibodies (1∶10000).

### Immunofluorescence Assay (IFA)

To examine the localization of GlEB1 in *G. lamblia* expressing HA-tagged GlEB1, the cells were attached to glass slides coated with L-lysine in a humidified chamber. The attached cells were fixed with chilled 100% methanol at −20°C for 10 min, and permeabilized with PBS/0.5% Triton X-100 for 10 min. After a 1 h-incubation in blocking buffer (PBS, 5% goat serum, and 3% BSA), the cells were reacted overnight with rat anti-GlEB1 polyclonal antibodies (1∶400) [13] and mouse anti-HA antibodies (1∶50; Sigma). Following three 5 min-washes with PBS, the cells were incubated with AlexaFluor 555-conjugated anti-rat IgG (1∶250; Molecular Probes) and AlexaFluor 488-conugated anti-mouse IgG (1∶200; Molecular Probes) at 37°C for 1 h. Slides were mounted with VECTASHIELD anti-fade mounting medium with 4′,6-diamidino-2-phenylindole (DAPI; Vector Laboratories). They were then observed with an Axiovert 200 fluorescent microscope (Carl Zeiss).

To determine the intracellular location of GlEB1 in dividing cells, trophozoites were treated with aphidicolin for 6 h, and released for 3 h. They were treated with anti-HA antibodies (1∶100) or anti-Glγ-tubulin antibodies (1∶100). In addition, encysting cells were also reacted with anti-HA antibodies to monitor GlEB1 localization during encystation. As a control to ensure the formation of cysts via an *in vitro*-encystation experiment, the encysting cells were also treated with anti-GlCWP1 antibodies (1∶100) [22]. These cells were then reacted with tetramethylrhodamine isothiocyanate (TRITC)-conjugated anti-rat IgG antibodies (1∶200; Jackson ImmunoResearch Lab).

### Synchronization of *Giardia* Trophozoites with Aphidicolin and Flow Cytometry Analysis

Synchronization of *Giardia* trophozoites was performed as described previously, with some modifications [23]. To summarize, 2×10^4^ cells were cultured in TYI-S-33 medium for 24−30 h until they reached 70% confluence. Aphidicolin (Sigma) was added to culture medium at a final concentration of 5 µg/mL, and then incubated for 6 h. The culture media was replaced with fresh medium, and cultured up to 6 h. Since the aphidicolin added to the culture was prepared in dimethyl sulfoxide (DMSO), control *Giardia* cultures were treated with 0.05% DMSO, showing that DMSO did not affect *G. lamblia* at that concentration. Synchronized cells were analyzed for their DNA content via flow cytometry, as described previously [24]. The DMSO-treated and aphidicolin-treated cells were placed on ice for 20 min, and harvested by 10 min centrifugation at 3000 rpm. The harvested cells were washed twice with 2 ml of HEPES-buffered saline (150 mM NaCl, 5 mM KCl, 1 mM MgSO_4_, and 10 mM HEPES, pH 7.4). The cells were resuspended in 300 µL of HEPES-buffered saline, and 700 µL of ice-cold 100% EtOH was then added drop by drop with a gentle vortex. Fixed cells were rinsed in 50 mM Na citrate (Sigma), and then treated in a 0.5 mL of 50 mM Na citrate containing 2.5 µg/mL RNase A for 30 min at 37°C. The cells were stained with propidium iodide (10 µg/mL; BD Pharminogen) in 50 mM Na citrate for 30 min at 4°C, and analyzed by a Beckman Coulter EPICS XL flow cytometer, and the data were analyzed with ModFit LT software (Becton Dickinson).

### Knockdown of GlEB1 Expression Using Morpholinos

The expression of GlEB1 was decreased by knockdown experiment as described [25]. Briefly, 25-mer morpholinos was designed to target the first 25 bases open reading frame of the GlEB1 (Table 2; Gene Tools). Another morpholinos identical to the GlEB1 morpholinos except 5 mispairs (Table 2) was used as a control. Lyophilized morpholinos were added to 5×10^6^ cells in 0.3 mL of medium at a final concentration of 100 µM. As a negative control, an equal volume of sterile water was added to the cells. After electroporation, cells were grown for 48 or 72 h, and then analyzed for expression of GlEB1 by Western blot and IFA as described above.

### Measuring Mitotic Index of *G. lamblia* Trophozoites

With *G. lamblia* trophozoites treated with water, mispair anti-EB1 morpholinos, or anti-EB1 morpholinos, the ratio of cells with 2 nuclei to those with 4 nuclei was determined to monitor mitotsis as described [26]. The cells were attached on coverslips were fixed with methanol for 5 min and air-dried. The cells were then mounted in VECTASHIELD anti-fade mounting medium with DAPI. The numbers of cells with 4 nuclei or 2 nuclei were counted in a total of 200 cells per each condition.

### Expression and Purification of rGlEB1 Proteins

Full-length rGlEB1 was expressed in *Escherichia coli* and purified as described previously [13]. In addition, the DNA fragment containing the GlEB1 ORF was dissected into two parts. The 5′-region of *eb1* (552 bp) was amplified from the genomic DNA of *Giardia* WB by PCR using the primers, EB1-full-ERI-F and EB1-CHD-R (Table 2), and the 3′-region of *eb1* (474 bp) was amplified with another set of primers, EB1-EBD-F and EB1-full-XhoI-R (Table 2). The resultant *eb1* DNA fragments were cloned into pET21b (Novagen) to generate overexpression plasmids, pET21b-EB1-CHD and pET21b-EB1-EBD, for the truncated rGlEB1 polypeptides. These rEB1 polypeptides were expressed in *E. coli* BL21 (DE3) with 0.5 mM IPTG at 30°C for 3 h, and purified using a TALON metal affinity chromatography as described by the manufacturer (Clontech).

### Microtubule Binding Assay

The binding of rGlEB1 to polymerized MTs was observed *in vitro* using the microtubule binding protein spin down assay kit BK029 (Cytoskeleton). Briefly, MTs were assembled from 100 µg of pure tubulins (isolated from bovine brain; Cytoskeleton) in 20 µL of PEM [80 mM piperazine-N,N′-bis(2-ethanesulfonic acid), pH 6.8, 1 mM EGTA, and 1 mM MgCl_2_] in the presence of 1 mM GTP and 5% glycerol at 35°C for 20 min, and immediately stabilized in 200 µL of warm PEM-20 µM taxol (Cytoskeleton). Various amount of MTs (0.5−2 µM tubulin) were incubated with 10 µg of rGlEB1 (0.38–0.7 µM) in a total volume of 50 µL at room temperature for 40 min. The reaction mixtures were then centrifuged through a 50% glycerol cushion-PEM-taxol mixture at 100000xg at 25°C for 40 min using an ultracentrifuge (Hitachi Koki), and the supernatant and pellet fractions were then resolved on SDS-PAGE.

Protein bands, the pellet fraction and the supernatant fraction, were quantified using Multi Gauge V3.0 software (Fujifilm) within the linear signal intensity range, which was determined using standard curves of rGlEB1. The fraction of rGlEB1 bound to MTs was plotted against the concentration of MT included in each reaction. To determine the apparent K_d_ of rGlEB1 for MTs, the data from three independent experiments were fitted by the equation Y = (B_max_+X)/(K_d_+X) using SigmaPlot version 9.0 (Systat software). Y is the concentration of rGlEB1 partitioning to the pellet with the MTs whereas B_max_ is the maximal fraction of rGlEB1 in rGlEB1-MTs complex. K_d_ is the dissociation constant where X is the concentration of MT used for each reaction.

### Size Exclusion Chromatography (SEC)

Full-length or truncated rGlEB1 protein (about 800 µg) was diluted with PBS on ice before loading onto a Superdex 75 10/300 GL (GE Healthcare). Eluted fractions were collected at a volume of 500 µL, and then separated by SDS-PAGE. Size control proteins of 75, 29, 14, and 6 kDa were loaded onto the same SEC. To examine an involvement of disulfide bond in dimerization of GlEB1, eluted proteins from SEC, were incubated with 100 mM DTT for 10 min prior to being separated upon SDS-PAGE.

### Site-directed Mutagenesis of the *eb1* Gene

To determine the role of the disulfide bond between the two EB1 monomers, cysteine residues at amino acid positions #13 and #46 were mutagenized to serine. Using the EB1 overexpression plasmid, pET21b-EB1-full, as a template, mutant DNA containing the mutation at amino acid position #13 was produced by PCR using primer EB-C13S-21b-F and EB1-full-XhoI-R (Table 2), resulting in a mutant EB1 DNA fragment containing the conversion of cysteine into serine at amino acid position #13. The expression plasmid pET21b-EB1-C46S carrying a mutation at cysteine position #46 of GlEB1 was made by PCR using primers EB1-full-ERI-F and EB-C46S-21b-R (Table 2), and the other mutant DNA carrying mutation at the same amino acid was also made by PCR using primers EB-C46S-21b-F and EB1-full-XhoI-R.

### Complementation Assay

*S. cerevisiae* strains (Table 1) were grown at 25°C in a synthetic complete (SC) dropout medium [0.17% yeast nitrogen base, 0.5% ammonium sulfate, 2% glucose, and 0.06% dropout mix lacking histidine (H), tryptophan (T) and uracil (U) (Clontech)]. The following ingredients were added to the medium at the indicated concentrations: H, 0,02; T, 0.02; and U, 0.02 mg/mL.

Two control plasmids, pZhu19 and pZhu20 [13], were used to complement GlEB1 and yeast Bim1p into YSK1134, the *bim1*Δ strain, respectively. An additional plasmid pRS426+P_GAL1–10_ EB1-C13S expressing mutant GlEB1 with C13S change was constructed by cloning 717 bp DNA fragment into SalI/SacII site of pRS426+P_GAL1–10_ and transformed into YSK1134.

Complementation assays were performed as described [13]. Briefly, yeast cells were synchronized at G1/S by incubation with 0.2 M hydroxyurea (HU; Sigma) for 4 h and released by washing several times with distilled water. To induce the expression of genes under the *GAL1-10* promoter, yeast cells were transferred to the SC dropout medium with 2% raffinose instead of glucose for 16 h, and then incubated with 0.2 M HU and 2% galactose/1% raffinose for 4 h. Following 1 min-fixation in 70% ethanol and staining with 1 µg/mL DAPI, the cells were observed with a 100 X objective on an Axiopvert 200 (Zeiss), and the images were captured with an Axiocam MRc camera using AxioVision software (Zeiss).

## Results

### Construction of *G. lamblia* Trophozoites Expressing HA-tagged GlEB1

**Figure 1:**
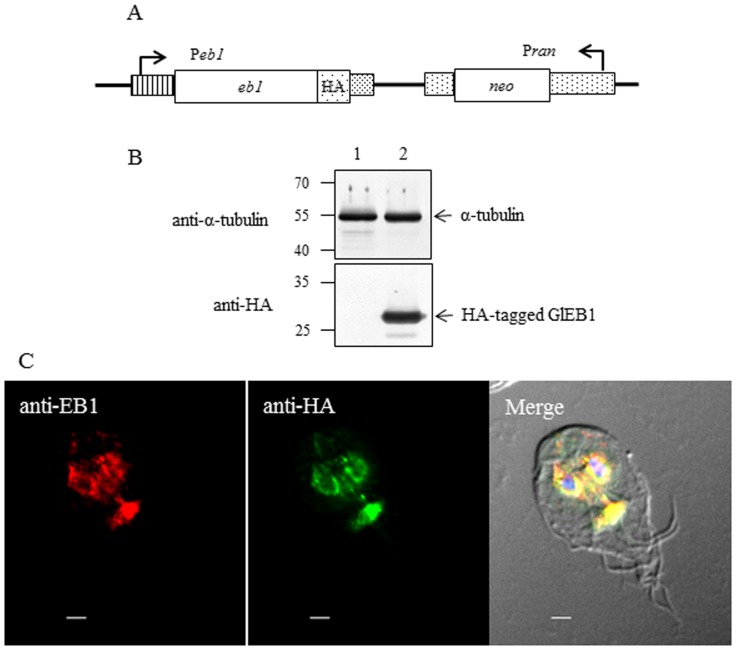
Expression and localization of GlEB1 in *G. lamblia*-expressing HA-tagged GlEB1. (A) Schematic diagram of plasmid pNLop2-eb1-HA-GItetR. GlEB1 is expressed as a HA-tagged form from its own promoter, P*eb1*. Transfected trophozoites are selected by G418 resistance conferred by the *neo* gene expressed by the P*ran* promoter, a strong promoter of the *ras*-related nuclear protein gene. As a control, *Giardia* trophozoites were also transfected with pNLop2-GItetR. (B) Western blot analysis to examine the expression of HA-tagged GlEB1. Extracts were prepared from *G. lamblia* containing pNLop2-GItetR (lane 1), or pNLop2-eb1-HA-GItetR (lane 2), and incubated with monoclonal mouse anti-HA antibodies (1∶1000) at 4°C overnight. Membranes were incubated in stripping buffer, and then reacted with polyclonal rat antibodies specific to α-tubulin of *G. lamblia* (1∶10000). (C) Localization of GlEB1. *G. lamblia* expressing HA-tagged GlEB1 was reacted with rat anti-GlEB1 polyclonal antibodies (1∶400) and mouse anti-HA (1∶50). The cells were then incubated with AlexaFluor 555-conjugated anti-rat IgG (1∶250) and AlexaFluor 488-conugated anti-mouse IgG (1∶100). Slides were mounted with VECTASHIELD anti-fade mounting medium with DAPI, and then observed with an Axiovert 200 fluorescent microscope. The scale bars are 2 µm.

End-binding 1 (EB1) protein is one of the +TIPs that connect MT spindles to the kinetochores and modulates MT stability [27]. In this study, functional analysis was performed on the only EB1 homologue in *G. lamblia*. An *E. coli-G. lamblia* shuttle plasmid was constructed to contain the DNA fragment encoding the promoter and ORF of GlEB1, from which EB1 is expressed as a fused form in frame with the HA epitope (Figure 1A). Upon electroporation and selection by G418 resistance, *G. lamblia* trophozoites carrying this plasmid were established and used for further investigation. These trophozoites were then examined for the expression of a chimeric EB1 tagged with an HA epitope by Western blot using anti-HA antibodies (Figure 1B). *G. lamblia* trophozoites carrying the vector plasmid without the *eb1* DNA fragment were also constructed, and included in the Western blot with anti-HA antibodies. As expected, the immunoreactive protein band was present in *Giardia* carrying the HA-tagged GlEB1-expressing plasmid, but absent in the control trophozoites. IFAs of *Giardia* trophozoites carrying the HA-tagged GlEB1-expressing plasmid were performed using two different antibodies, anti-HA antibodies and anti-GlEB1 antibodies (Figure 1C). Incubation of these trophozoites with anti-HA antibodies revealed fluorescence at the nuclear membranes and median bodies. IFA using anti-GlEB1 also demonstrated fluorescence staining at the same positions in these transgenic *Giardia* trophozoites as shown in IFA using anti-HA antibodies.

### Determination of Expression Levels and Localization of GlEB1 at Different Stages of the *Giardia* Life Cycle

**Figure 2:**
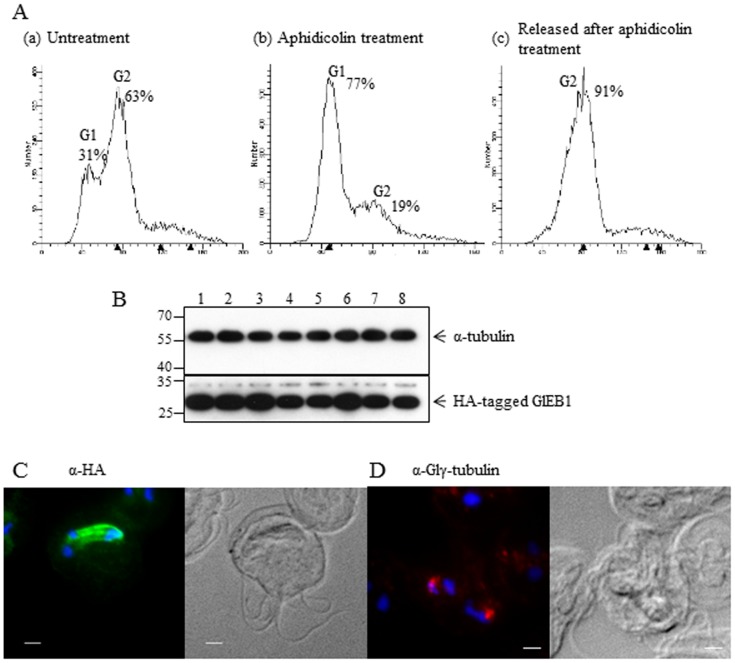
Expression and localization of GlEB1 in synchronized *Giardia* carrying pNLop2-eb1-HA-GItetR. (A) Flow cytometric analysis of *Giardia* trophozoites treated with 0.05% DMSO (a), trophozoites arrested with 5 µg/mL aphidicolin for 6 h (b), and trophozoites arrested with aphidicolin and released for 3 h (c). (B) Western blot analysis of synchronized cells using anti-HA (1∶2000). Lane 1, *Giardia* cultures treated with 0.05% DMSO; lane 2, aphidicolin-treated cells; and lane 3-8, cells released from aphidicolin treatment up to 6 h. The amount of α-tubulin was also monitored in these cells using anti-Glα-tubulin (1∶10000). (C) Localization of GlEB1 in trophozoites at the G2 phase. G2-phase cells were prepared by 6 h-treatment with aphidicolin and following 3 h-release. The cells were reacted with anti-HA (1∶100) for observation under fluorescence microscopy. As a marker for G2-phase cells, the cells were treated with anti-Glγ-tubulin (1∶100). The scale bar is 2 µm.

Limited information is available on the expression pattern of GlEB1 in *G. lamblia*. In a previous study, we reported that the intracellular level of GlEB1 is not altered during encystation [13]. In order to examine the expression level of GlEB1 in different stages of the cell cycle, we obtained synchronized trophozoites using *Giardia* carrying the GlEB1 expression plasmid (Figure 2A). Flow cytometry analysis (FACS) of their DNA content indicated that *Giardia* trophozoites without any treatment were comprised of cells at G1 phase (31%) and at G2 phase (63%). After 6 h-treatment with aphidicolin, the majority of *Giardia* trophozoites (77%) were arrested at the G1 phase. The aphidicolin-treated cells were transferred to drug-free medium in order to be released into G2 phase. Released cells showed a DNA pattern that is typical of cells at G2 phase (91%).

*Giardia* cells expressing HA-tagged GlEB1 were harvested at various stages, trophozoite without drug treatment, aphidicolin-treated cells, and cells released from growth arrest at various times, ranging from 1 to 6 h. These extracts were analyzed by Western blots to determine intracellular levels of GlEB1 at different stages of *Giardia* growth, indicating that GlEB1 expression does not vary significantly by cell cycle (Figure 2B). Western blot of these extracts with anti-Glα-tubulin antibodies served as a loading control.

*Giardia* cells at G2 phase, released cells from aphidicolin treatment, were examined by IFAs using anti-HA antibodies (Figure 2C). Mitotic cells were frequently observed with mitotic spindles labeled with fluorescence, indicating that GlEB1 was localized to the mitotic spindles of the mitotic cells at anaphase. IFA of these cells with anti-Glγ-tubulin antibodies showed fluorescent labeling at the two basal bodies during anaphase, confirming that they are mitotic cells (Figure 2D).

**Figure 3:**
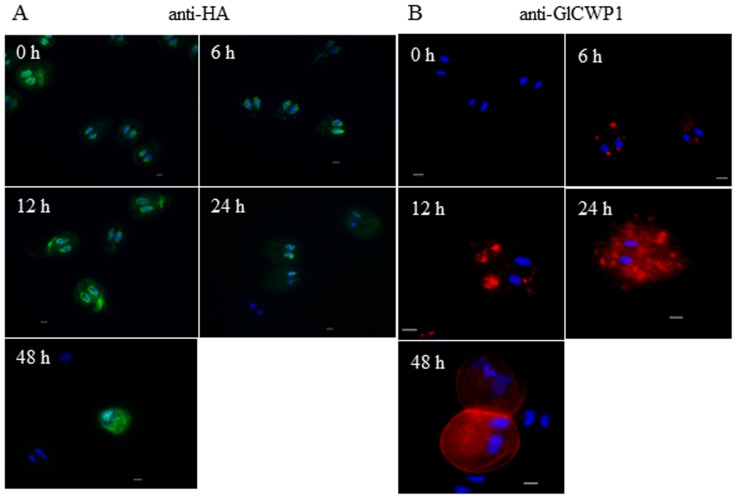
Localization of GlEB1 in *G. lamblia* during encystation. *Giardia* trophozoites and encysting cells were prepared at various time-points of post-induction of encystation. (A) Fixed cells were serially reacted with mouse anti-HA antibodies (1∶100) and AlexaFluor 488-conjugated anti-mouse IgG (1∶200). (B) As a control for encystation, a separate set of trophozoites and encysting cells was reacted with anti-GlCWP1 antibodies (1∶100). The scale bar is 2 µm.

The intracellular level of GlEB1 was found to be constitutive during encystation [13]. In this study, we examined the possibility that localization of EB1 was altered during encystation by IFA of *Giardia* expressing HA-tagged GlEB1 with anti-HA antibodies (Figure 3A). Up to 24 h-after induction to encystation, GlEB1 was mainly found at the nuclear membrane in the most of the *Giardia* cells, whereas some of the cells showed fluorescent labeling of the median bodies. In cysts at 48 h induction, fluorescence was absent in the nuclear membranes and dispersed in the cytoplasm of the cells. As a control for encystation process, intracellular location of CWP1 was also examined in trophozoites as well as encysting cells (Figure 3B). CWP1 was barely detected in trophozoites, and later found in encystation-specific vesicles in the encysting cells. Localization of CWP1 in the cyst walls was distinct in the cysts at 48 h-after induction of encystation. These results showed that the location of GlEB1 at the nuclear membrane and median bodies was changed into a condensed pattern in the cytoplasm of cysts.

### Effect of GlEB1 Knockdown in Mitosis of *G. lamblia*

To define the role of EB1 in *G. lamblia*, we designed a morpholinos, anti-EB1, which can block translation of GlEB1 mRNAs (Table 2). As a control, another morpholinos, mispair anti-EB1, was made and used to transfect into *G. lamblia* trophozoites by electroporation (Table 2). In addition, a set of *G. lamblia* was treated with sterile water instead of morpholinos.

**Figure 4:**
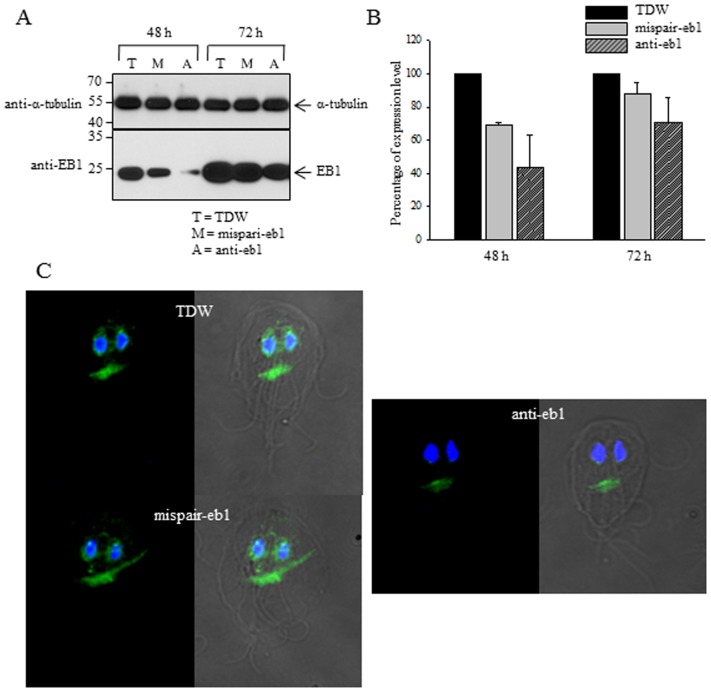
Morpholinos-mediated knockdown of GlEB1 expression in *G. lamblia*. *Giardia* trophozoites were collected at 48 and 72 h after electroporation with water (W), mispair anti-EB1 morpholinos (M), or anti-EB1 morpholinos (A). (A) Extracts of these cells were analyzed by Western blots using anti-EB1 antibodies or anti-α-tubulin antibodies. (B) Relative expression of GlEB1 in cell extracts treated with mispair anti-EB1 morpholinos (M), or anti-EB1 morpholinos. (C) The cells were attached to glass slides coated with L-lysine in a humidified chamber. The attached cells were fixed with chilled 100% methanol at -20°C for 10 min, and permeabilized with PBS/0.5% Triton X-100 for 10 min. After a 1 h incubation in blocking buffer (PBS, 5% goat serum, and 3% BSA), the cells were reacted overnight with mouse anti-GlEB1 polyclonal antibodies (1∶100). Following three times 5 min-washes with PBS, the cells were incubated with AlexaFluor 488-conugated anti-mouse IgG (1∶200; Molecular Probes) at 37°C for 1 h. Slides were mounted with VECTASHIELD anti-fade mounting medium with DAPI. They were then observed with an Axiovert 200 fluorescent microscope.

Cell extracts prepared from these cells at 48 h and 72 h post-transfection were monitored for their intracellular levels of EB1 by Western blot using anti-GlEB1 antibodies (Figure 4A). Both cells treated with mispair anti-EB1 or anti-EB1 morpholinos showed decreased amount of GlEB1 at 48 h post-transfection to 69% and 43%, respectively (Figure 4B). Amount of GlEB1 was restored to 88% and 70% of that of cells treated with water in cells at 72 h post-transfection. Decreased level of GlEB1 was also confirmed by IFA of *G. lamblia* cells treated with sterile water, the mispair anti-EB1, or anti-EB1 morpholinos after 48 h (Figure 4C). GlEB1 was found in nuclear membrane and median bodies in cells treated with water or mispair anti-EB1 morpholinos. In *G. lamblia* transfected with anti-EB1 morpholinos, GlEB1 was barely detected in nuclear membranes. Clear staining of median bodies were observed in *G. lamblia* cells treated with anti-EB1 morpholinos, but at lower levels than in cells treated with water or mispair anti-EB1 morpholinos.

**Figure 5:**
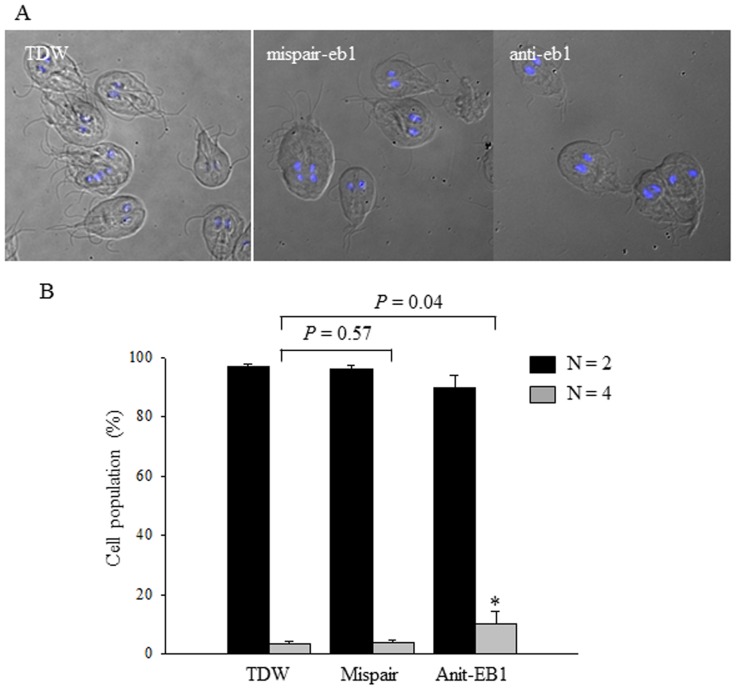
Mitotic index of *Giardia* trophozoites transfected with water, mispair anti-EB1 morpholinos, or anti-EB1 morpholinos. (A) Upon staining with DAPI, the cells were counted for those with two nuclei or four nuclei. (B) *Giardia* trophozoites transfected with water, mispair anti-EB1 morpholinos, or anti-EB1 morpholinos, were counted for the numbers of their nuclei, and then indicated in the graph as percentages. The percentage of cell population with a two or four nuclei was significantly different each conditions by the Student's *t*-test. Data with *p*-value of <0.05 are indicated with an asterisk. Standard deviations were derived from three independent experiments. The percentage of cell population was counted in a total of 200 cells per each condition and measured in triplicates for each sample.

Effect of low expression of GlEB1 was determined by measuring mitotic indexes, which is portions of cells with four nuclei (Figure 5). Upon staining with DAPI, the cells were counted for those with two nuclei or four nuclei (Figure 5A). About three percentages of cells contained 4 nuclei when they were treated with water or mispair anti-EB1 morpholinos. The percentage of cells with four nuclei was increased to 10% upon transfection with anti-EB1 morpholinos (Figure 5B). This result indicates that GlEB1 plays some role in mitosis of *G. lamblia*.

### Characterization of the Functional Domain for MT-binding of GlEB1

GlEB1 contains three putative domains: a CH domain (#21-#101), a linker (#102-#184), and an EB1 domain conserved in other EB1 homologues (#185-#238). Interestingly, GlEB1 lacks the C-terminal acidic tail ended with the EEY/F motif. In addition to the full-length rGlEB1, rGlEB1_1–238_, two truncated rGlEB1 proteins, rGlEB1_1–184_, and rGlEB1_102–238_, were expressed in *E. coli*, and purified using affinity chromatography. rGlEB1_1–184_ contains the CH domain and a linker region whereas rGlEB1_102–238_ has the linker and the EB1 domain.

**Figure 6:**
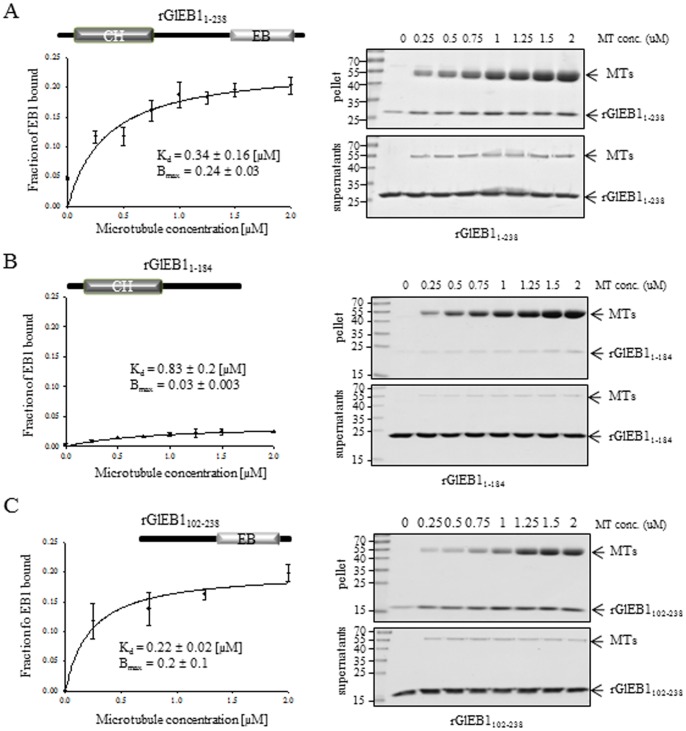
EB1 binding to polymerized MTs *in vitro*. An equal amount of rGlEB1 was incubated with an increasing amount of taxol-stabilized bovine microtubules (0−2 µM). rGlEB1 bound to MTs was separated from the unbound fraction by ultracentrifugation, and the amount of rGlEB1 in the supernatant and pellet was quantified. The graph shows the averaged data of three independent experiments. (A) Binding of full-length rGlEB_1–238_, (B) binding of truncated rGlEB1_1–184_, and (C) binding of truncated rGlEB1_102–238_.

The MT-binding region in GlEB1 was identified by testing the three rGlEB1 proteins in a sedimentation assay with taxol-stabilized MTs (Figure 6). Full-length rGlEB1 showed a significant affinity for the MTs, with a dissociation constant (K_d_) of 0.34 µM and 24% of the added rEB1 was precipitated with the MTs. On the other hand, rGlEB1_1–184_ containing the CH domain and the linker showed significant attenuation in MT-binding, resulting in a K_d_ of 0.83 µM and a B_max_ of 0.03. Interestingly, rGlEB1_102–238_ containing the linker and the EB1 domain demonstrated a comparable MT-binding ability to that of the full-length rEB1 in this assay (K_d_ = 0.22 µM, B_max_ = 0.2).

### Characterization of a Functional Domain for the Dimerization of GlEB1 Protein

**Figure 7:**
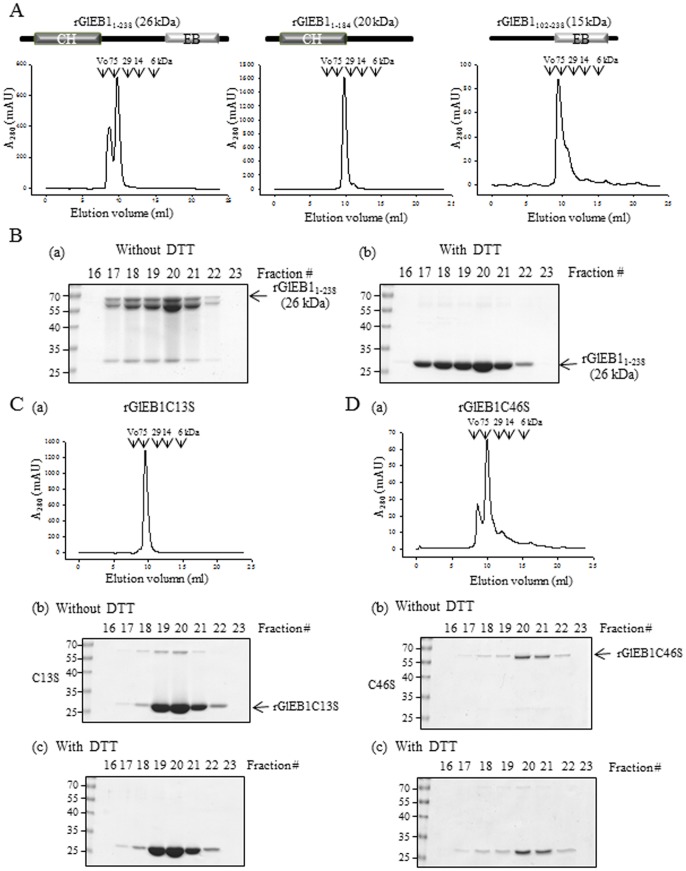
Size exclusion chromatography (SEC) of rGlEB1 proteins. (A) SEC of three rGlEB1 proteins, full-length rGlEB1_1–238_, truncated rGlEB1_1–184_, and truncated rGlEB1_102–238_. CH represents the putative calponin homology domain whereas EB indicates the EB1-specific domain. Each of the three rGlEB1s was passed through a Superdex 70 10/300 GL column. Four size markers of 75, 29, 14, and 6 kDa were also passed through SEC and indicated in each elution graph. (B) SDS-PAGE of the SEC fractions of rGlEB1_1–238_, without any treatment (a) or with DTT treatment (b). (C) SEC profile of C13S mutant rGlEB1 (a), SDS-PAGE of the SEC fraction without DTT treatment (b), and SDS-PAGE of the SEC fraction with DTT treatment (c). (D) SEC profile of C46S mutant rGlEB1 (a), SDS-PAGE of the SEC fraction without DTT treatment (b), and SDS-PAGE of the SEC fraction with DTT treatment (b).

Each of these three rGlEB1 proteins was analyzed by size exclusion chromatography (SEC) to examine whether they are present as a dimeric form (Figure 7A). rGlEB1_1–238_ eluted as two overlapping peaks earlier than the marker protein of 29 kDa, indicating the dimeric formation of rGlEB1_1–238_. rGlEB1_1–184_ containing a CH domain and the linker, also eluted earlier than the 29 kDa marker protein, suggesting that this truncated protein is able to form dimers. In the case of rGlEB1_102–238_, they passed through the column as an elongated, dimeric peak, raising the possibility of conformational modification or multimeric formation of rGlEB1_102–238_. Elution of rGlEB1_1–184_ in a dimeric form from SEC is interesting in that this region is involved in MT-binding, but not in the dimerization of Bim1p, an EB1 homolog of budding yeast [5].

When the eluted fractions from SEC were analyzed via SDS-PAGE, rGlEB1_1–238_ appeared as a dimer and became monomer with the addition of dithiothreitol (DTT) (Figure 7B). This result suggests that intermolecular disulfide bond(s) are involved in the formation of GlEB1 dimers. The amino acid sequence of GlEB1 revealed two cysteine residues at amino acid position #13 and #46. Based on the location of these cysteine residues, rGlEB1_1–184_ was also examined, in which dimerization occurs through intermolecular disulfide bond(s). rGlEB1_1–184_ was present as 40 kDa without DTT, and became 20 kDa upon incubation with DTT. As expected, rGlEB1_102–238_ appeared as a monomer regardless of the presence of DTT (data not shown).

Two cysteine-mutant rGlEB1 variants, C13S and C46S, were analyzed by SEC (Figures 7C 7D, respectively). C13S-mutant rGlEB1 eluted as a single peak in dimeric form, and appeared as a 26 kDa band on SDS-PAGE, regardless of DTT. C46S-mutant rGlEB1 eluted as two overlapping peaks earlier than the marker protein of 29 kDa, and appeared as a dimer on SDS-PAGE without DTT. This experiment indicated that dimer formation of *Giardia* EB1 occurs via two different mechanisms: the interaction between the EB1 domains and intermolecular disulfide bond formation of two EB1 monomers. Mutant rGlEB1, containing both C13S and C46S conversions showed an elution pattern identical to that of the C13S-mutant rGlEB1 (data not shown).

### Complementation of Yeast *BIM1* Mutant with C13S mutant GlEB1

To examine whether the putative EB1 protein of *G. lamblia* plays a role in controlling MT dynamics, we used a budding yeast system that is amenable to various genetic approaches. In addition to the positive controls for complementation for giardial *eb1* and the *BIM1* gene of *S. cerevisiae*, mutant *eb1* gene encoding mutant GlEB1 with C13S was cloned into the same yeast expression system and transformed into YSK1134 (*bim1Δ* mutant).

**Figure 8:**
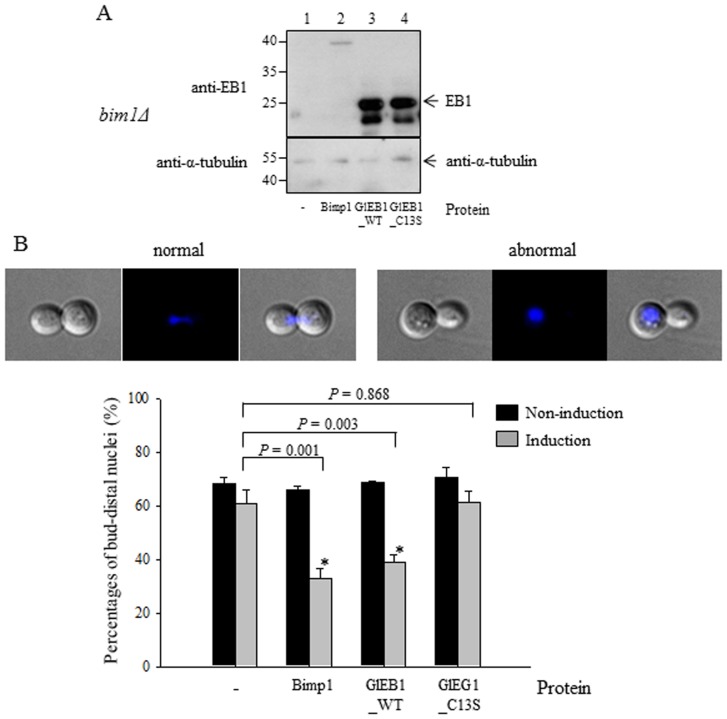
Complementation of wild type or C13S mutant GlEB1 in the *BIM1* knock-out mutants of *S. cerevisiae*. (A) Expression of wild type or C13S mutant GlEB1 in yeast. A plasmid, pRS426+P_GAL1–10_, serves as a vector control, which has a galactose/raffinose-inducible promoter functioning in budding yeast. For pZhu19, the *eb1* gene of *G. lamblia* was inserted under P_GAL1–10_, whereas plasmid pZhu20 containing the *BIM1* gene, a yeast homologous gene for *eb1*, serves as a positive control for complementation. In addition, mutant *eb1* gene with C13S change was expressed in yeast. The expression of giardial EB1 protein in various yeast cells carrying a vector plasmid or one of the complementation plasmids. Extracts of various yeast cells were analyzed by a Western blot using EB1-specific antibodies. (B) The percentage of Bim1p- or wild type GlEB1-complemented strains with bud-distal nucleus under induced conditions was significantly different from that of control strain carrying vector plasmid. On the other hand, the percentage of C13S mutant–complemented strain with bud-distal nucleus was not statistically different from that of control yeast carrying vector plasmid. The percentage of complemented strains with a bud-distal nucleus under inducing conditions was significantly different from that of the control strain carrying pRS426+P_GAL1–10_ under inducing conditions by the Student's *t*-test. Data with *p*-value of <0.01 are indicated with an asterisk. Standard deviations were derived from three independent experiments. The percentage of abnormal cells with biased nucleus distribution was measured in triplicates for each sample.

The expression of these *G. lamblia* EB1 proteins in the yeast transformants was examined by Western blot analysis using anti-GlEB1 antibodies (Figure 8A). The *bim1Δ* cells carrying only pRS426+P_GAL1–10_ (negative controls) or yeast *BIM1* gene did not show any immunoreactive protein. Only the mutant yeast strains carrying wild type *eb1* gene or mutant *eb1* gene showed two immunoreactive bands.

We examined the nucleus position in YSK1134 (*bim1Δ*) cells expressing giardial EB1 proteins or yeast Bim1p in comparison with the control, YSK1134 carrying pRS426+P_GAL1–10_. These yeast cells were grown in the presence of glucose (as a non-induced condition) or in the presence of galactose and raffinose (as an induced condition) and number of the yeast cells with bud-distal nuclei was determined under both conditions (Figure 8B). About sixty percent of *bim1Δ* cells carrying pRS426+P_GAL1–10_ showed defects in nucleus positioning. Under induced conditions, the percentage of cells with a bud-distal nucleus were decreased to 38% and 40% in the *bim1Δ* cells carrying pZhu20 (wild type *BIM1* gene) and pZhu19 (wild type *eb1* gene). indicating a partial, but significant complementation of the *bim1Δ* yeast cells with *Giardia eb1* gene. On the other hand, the *bim1Δ* cells carrying pRS426+P_GAL1–10_ EB1-C13S with C13S mutant EB1 maintained a similar level of cells with a bud-distal nucleus (62%). These results demonstrate that the EB1 homolog of *G. lamblia* can function similarly to Bim1p in the budding yeast and cysteine residue #13 of GlEB1 plays a role in this process.

## Discussion

Understanding the role of EB1 is important in revealing the mechanism of how *Giardia* divides with a coordinated distribution of its organelles and cytoskeletal structures. In this study, the function of GlEB1 was assessed by monitoring the expression and localization of GlEB1 at various phases of the cell cycle (Figures 2B and 2C, respectively) and during encystation (Figure 3A). A study using green fluorescent protein-tagged GlEB1, demonstrated its localization at flagellar tips and median bodies, and suggested that it is also located at mitotic spindles [12]. IFA demonstrated that GlEB1 is localized to the nuclear membranes, median bodies, and axonemes of the trophozoites [13]. *Giardia* cells arrested at the G1 and G2 phases did not show a dramatic change in intracellular GlEB1 levels (Figure 2B). Western blot analysis showed that the intracellular amount of GlEB1 is constant in encysting cells as well as trophozoites [13]. These observations are consistent with information on the expression levels of EB1 in other organisms in which a constant amount of EB1 is maintained at any stage of cell division and cell differentiation [28].

It is likely that EB1 localization at the correct sites is critical for its proper function. As expected, in mitotic cells, localization of GlEB1 in mitotic spindles was clearly observed (Figure 2C). IFA using anti-Glγ-tubulin antibodies (Figure 2D) demonstrated its localization to the basal bodies of mitotic cells of *Giardia* at anaphase, which had been previously reported in *G. lamblia* [29]. Intracellular localization of GlEB1 in the encysting cells was in the nuclear membrane and median bodies (Figure 3A). GlEB1 is localized to the cytoplasm in a more dispersed pattern in the cyst form. This observation needs to be explained through an extensive investigation of cyst structures using more cell markers.

EB1 proteins are the most conserved and ubiquitous +TIPs in eukaryotes [30]. Putative amino acid sequences of GlEB1 demonstrated the presence of a CH domain, a linker, and an EB1 domain (Figure 6 and 7). Interestingly, GlEB1 lacks the EEY/F motif which is conserved in other eukaryotes [6]. A previous study demonstrated that rGlEB1 is precipitated with polymerized MTs *in vitro* [13]. In this study, the role of the two domains in GlEB1 was assessed using two truncated forms of rGlEB1, rGlEB1_1–184_ with the CH domain and linker, and rGlEB1_102–238_, containing a linker and EB1 domain. Unexpectedly, rGlEB1_1–184_ with the CH domain showed dramatic attenuation in its MT-binding ability. The CH domain is known to be involved in MT binding in other organisms [31]. However, budding yeast Bim1p containing only the N-terminal CH domain was also found to be defective in MT binding due to its inability to form dimers [5]. Loss of the MT-binding ability of rGlEB1_1–184_ containing the CH domain and a linker did not occur because this truncated rGlEB1_1–184_ is able to form dimers in an EB1 domain-independent manner (Figure 7A). We cannot rule out the possibility that the conformation of this truncated rGlEB1_1–184_ was modified as it is impertinent to MT binding. Interestingly, rGlEB1_102–238_ with the EB1 domain and linker demonstrated equivalent binding to MTs to that of full-length rGlEB1. It is contrary that the N-terminal-truncated rBim1p containing the EB1 domain showed dramatic defect in MT-binding assays [5].

Using these three rGlEB1 proteins, we examined whether GlEB1 was also present as a dimer (Figure 7A). SEC analysis indicated that both full-length rGlEB1_1–238_ and rGlEB1_1–184_ with the CH domain were found as dimers. rGlEB1_102–238_ carrying the EB1 domain eluted earlier than 29 kDa, the expected size of the dimer. This can be explained by rGlEB1 having a conformational change or modification resulting in earlier elution in SEC analysis. Otherwise, this rGlEB1 may have a tendency to form multimer.

Interestingly, full-length rGlEB1 eluted as two peaks, an earlier smaller peak and a later major peak (Figure 7A). SDS-PAGE of the eluted proteins yielded two protein bands larger than the monomer, which became 26 kDa after DTT treatment, indicating the presence of a disulfide bond in the GlEB1 dimer. *In silico*-analysis of GlEB1 amino acid sequences revealed that two cysteine residues are present at amino acid residues #13 and #46. EB1 proteins of other organisms (*S. cerevisiae*, *Homo sapiens*, and *Mus musculus*) have 3–4 cysteine residues; specifically 2–3 cysteines are present in the N-terminal portion of the protein. Formation of a disulfide bond between the EB1 monomers has not yet been reported in these organisms. Site-directed mutagenesis of these two cysteine residues clearly indicate that cysteine #13 is involved in intermolecular disulfide bonds between two monomer of EB1. Dimer formation of GlEB1 is mediated through an EB1 domain-mediated interaction, as suggested by the other system, which appeared as a major band in SEC. In addition, the earlier minor peak is derived from disulfide bond formation as it disappeared in rGlEB1 with C13S replacement. The presence of dimeric GlEB1, mediated by an intermolecular disulfide bond *in vivo* form should be examined in further investigation.

A comparison of GlEB1 with other EB1 sequences revealed C-terminal tail sequences ranging from 20 bases (*H. sapiens* and *M. musculus*) to 68–70 bases (*S. cerevisiae* and *Schizosaccharomyces pombe*). Along with the EB1 domain, these C-terminal tail sequences are known to be involved in interactions with other +TIPs, such as CLIP and p150 [32], [33]. Interestingly, GlEB1 has an unusually short C-terminal tail of four bases (EYHQ). In the case of human and murine EB1, the last three amino acids are EEY, which are involved in the interaction with +TIPs [34], [35]. In the case of the EB1 of budding yeast, the last three amino acids are ETF, which have similar biochemical properties to that of EEY. However, EB1 homologues of fission yeast and plants do not have the C-terminal tail sequence as well as GlEB1. An important question would be to identify the functional difference caused form the lack of these sequences in GlEB1. The cytoskeleton-associated protein-glycan-rich (CAP-Gly) domain of +TIPs is known to function in interaction with the C-terminal EEY sequence of EB1 and microtubules in mammalians and yeasts [36]. In contrast with GlEB1, two putative α-tubulins of *G. lamblia* (accession numbers GL50803_112079 and GL50803_103676) have a C-terminal tail sequence (MEEDDAY), which shows 4 conserved amino acids of the 6 consensus C-terminal tail, P/E/D-E/D-P/E/D-E-E/T-Y/F [36]. As interacting proteins with C-terminal EEY sequence, proteins having the CAP-Gly domain were searched in *Giardia* database, resulting in identification of two putative proteins, tubulin specific chaperone B (GL50803_5374) and tubulin specific chaperone E (GL50803_16535). Thus, it is likely that tubulin interacts with tubulin specific chaperon in *G. lamblia*, even though it should be experimentally proven.

Role of GlEB1 in mitosis was indirectly examined via complementation experiment using budding yeast system [13]. In this study, we examined the role of GlEB1 in *G. lamblia* via the knockdown experiments using morpholinos-based anti-sense oligonucleotides (Figures 4 and 5). At 48 h-post transfection, inhibition of EB1 expression was dramatic. Cells transfected with mispair anti-EB1 morpholinos, also showed decreased level of GlEB1. However, the cells transfected with anti-EB1 showed more dramatic reduction in GlEB1 expression as measured in Western blot analysis (Figures 4A and 4B) and IFA (Figure 4C). Significant increase of cells with 4 nuclei among anti-EB1 morpholinos-treated cells indicated that GlEB1 play an important role in mitosis of *G. lamblia* (Figure 5). In addition to mitotic index, we observed that size of median bodies was reduced in *Giardia* treated with anti-EB1 morpholinos (data not shown). This observation needs to be documented in further investigation. The complementation assay using the *BIM1* mutant *S. cerevisiae* indicated that GlEB1 can replace the function of Bim1p in the budding yeast and dimerization of GlEB1 via its 13rd cysteine residue is important (Figure 8B). Functional complementation between EB1 homologues of different organisms has been reported [37]. Human EB1 can substitute for the loss of *mal3^+^* gene product, the fission yeast EB1 homologue, as appeared suppression of its hypersensitivity to thiabendazole, the microtubule destabilizing drug.

This study provides an *in vitro* biochemical characterization of *Giardia* EB1 with respect to its MT-binding activity and dimerization. Dimer formation of GlEB1 was mediated by an interaction between the EB1 domains and an intermolecular disulfide bond. In addition, role of EB1 in division of *G. lamblia* was shown by the knockdown experiment.
